# Change of precipitation characteristics in the water-wind erosion crisscross region on the Loess Plateau, China, from 1958 to 2015

**DOI:** 10.1038/s41598-017-08600-y

**Published:** 2017-08-14

**Authors:** Xingkai Zhao, Zengyao Li, Qingke Zhu

**Affiliations:** School of soil and water conservation, Beijing Forestry University, Beijing, 100083 China

## Abstract

Precipitation plays an important and crucial role in processes in the water-wind erosion crisscross region of the Loess Plateau than in other parts of the region. We analyzed precipitation data and standardized precipitation index (SPI) at 14 representative synoptic stations from 1958 to 2015 used trend-free prewhitening, linear trend estimation, Spearman’s rho test, the Mann-Kendall trend test, the Mann-Kendall abrupt change test and rescaled range analysis. The following conclusions were drawn. First, the analysis of monthly precipitation at all stations suggested that precipitation during the rainy season (July, August, September), especially rain in July and August, exhibited a general decreasing trend, while both increasing and decreasing trends were observed in other months. Moreover, the annual precipitation of all stations continued to exhibit decreasing trends except Wuzhai. Erosive rainfall frequency in the rainy season and the annual scale was weakly reduced but erosive force of single rainfall has been enhanced. Second, the SPI exhibited different increasing degrees in winter, while decreasing trends were observed in other seasons. Additionally, the annual-scale SPI at most stations exhibited a stable and sustained downward trend. Therefore, this region is currently associated with a drought trend, and the drought degree will likely continue to increase.

## Introduction

Drought is a common natural disaster on the Loess Plateau in China. Additionally, the Loess Plateau is one of the most serious areas of erosion in China and even the world, and the main erosion forms are water erosion and wind erosion.

Precipitation is an important indicator that can be used to directly characterize climate drought. In studies that analyze climate drought and wetness, the commonly selected indices include the Palmer Drought Severity Index (PDSI)^1, 2^, the standardized precipitation index (SPI)^3, 4^, and the standardized precipitation evapotranspiration index (SPEI)^5, 6^. The SPI is a drought index recommended by the World Meteorological Organization (WMO)^7^. Studies of precipitation and dry/wet conditions on the Loess Plateau have been previously conducted. Notably, the characteristics of precipitation and air temperature on the Loess Plateau from 1961 to 2011 were analyzed by Sun *et al*.^8^ and Miao *et al*.^9^, and they found that the risks of severe floods and droughts on the Loess Plateau increased between 1961 and 2011. Zhang *et al*.^10^ analyzed the climate change characteristics in spring and summer on the Loess Plateau from 1971 to 2010. Zhao *et al*.^11^ and Li *et al*.^12^ used different methods to study the characteristics of precipitation and drought in the loess region of Northern Shaanxi Province, China. Liu *et al*.^13^ compared the effects of the SPI and SPEI in an analysis of the wet and dry characteristics of the Loess Plateau Region from 1957 to 2012, and they noted that the results of the two indices differed. Xu *et al*.^14^ found that the SPI was more appropriate than the SPEI in arid regions based on an analysis of drought on the Loess Plateau from 1961 to 2012.

Both water and wind erosion are important contributors for regional erosion and they can promote each other in the wind-water erosion crisscross region of the Loess Plateau. Thus, this crisscross region is an area of serious erosion issues on the Loess Plateau. To reduce the harm caused by erosion, the factors that influence erosion^15, 16^ and the ecological measures that prevent erosion, such as vegetation restoration^17–19^, have been studied in detail. Precipitation is the main limiting factor for vegetation restoration on the Loess Plateau^20, 21^, and both precipitation and temperature affect the growth of plants^22^.

Precipitation is closely related to hydraulic erosion^23^, especially when the daily precipitation is greater than 12 mm^24–26^, and a lack of precipitation can cause drought. Consequently, droughts often cause more severe wind erosion and accelerate land degradation. Therefore, in terms of erosion and drought, precipitation plays a more important and crucial role in the water-wind erosion crisscross region of the Loess Plateau than in other parts of the region. In the context of global climate change^27, 28^, it is necessary to analyze the regions with similar and precise geographical divisions obtain accurate conclusions. Comprehensive analyses of the precipitation and dry/wet characteristics of the wind-water erosion crisscross region can improve regional water resource planning and management, the local water use efficiency, the efficiency of vegetation restoration and regional environmental regulation; however, such analyses are currently lacking.

The main objectives of this study are as follows: (1) to identify the characteristics of precipitation in the water-wind erosion crisscross region on the Loess Plateau from 1958 to 2015; (2) to analyze the precipitation trends at different time scales based on sequential autocorrelation; and (3) to evaluate the wet/dry conditions of the water-wind erosion crisscross region on the Loess Plateau from 1958 to 2015 based on the SPI.

## Results

### Analysis of precipitation characteristics

**Figure 1:**
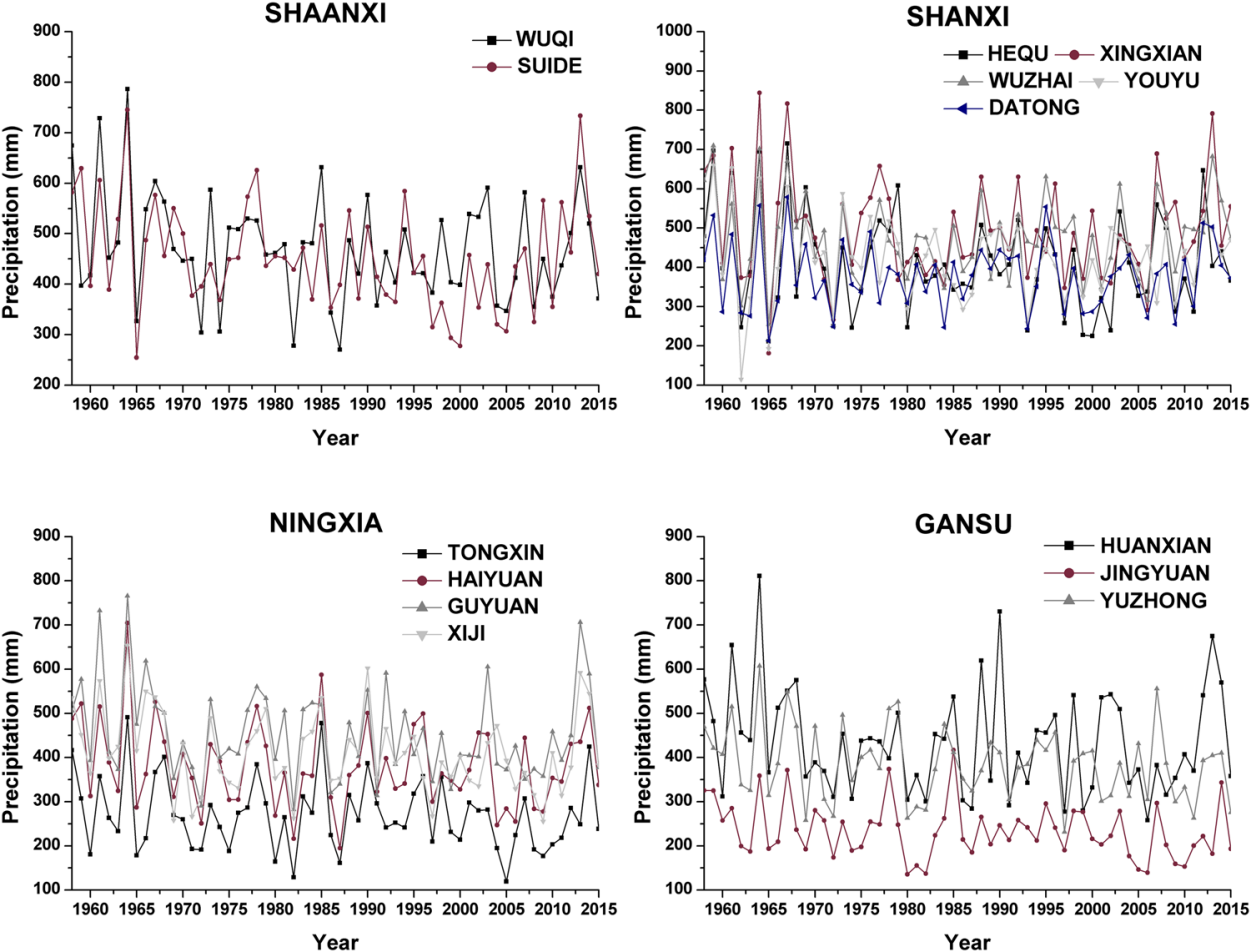
Time series of annual precipitation at each synoptic station.

Time series of annual precipitation collected at 14 synoptic stations in the study area from 1958 to 2015 (Fig. 1) and annual precipitation characteristics from each station (Table 1) show that precipitation exhibited a skewed distribution during the study period. The spatial differences in precipitation were obvious in the study area. Notably, the average annual precipitation at the 14 synoptic stations ranged from 234.59 (Jingyuan) to 493.28 mm (Xingxian), and the highest and lowest amounts of annual precipitation were 844.4 mm (Xingxian station in 1964) and 114.9 mm (Youyu station in 1962), respectively. The interannual variations in annual precipitation at 14 synoptic stations were also significant. The coefficients of variation (CVs) of annual precipitation were higher than 20% at the synoptic stations, and the highest value occurred at Hequ (31.00%), while the lowest value was 21.57% at Youyu.

**Table 1:** Annual precipitation characteristics for each synoptic station.

Station	Min (mm)	Max (mm)	Mean (mm)	Standard deviation (mm)	CV (%)	Skewness	Kurtosis
Wuqi	270.0	786.3	470.25	106.39	22.62	0.539	0.501
Suide	254.4	745.2	453.52	105.44	23.25	0.589	0.282
Hequ	211.3	715.3	410.12	127.14	31.00	0.588	−0.143
Xingxian	181.1	844.4	493.28	131.61	26.68	0.536	0.486
Wuzhai	252.5	709.6	474.15	107.75	22.73	0.167	−0.371
Youyu	114.9	659.8	422.65	104.52	24.73	−0.154	0.728
Datong	212.8	578.6	378.01	85.51	22.62	0.309	−0.395
Tongxin	119.4	490.8	270.09	80.44	29.78	0.691	0.365
Haiyuan	194.5	704.8	380.95	96.53	25.34	0.727	0.934
Guyuan	282.1	765.7	454.54	105.17	23.14	0.901	0.746
Xiji	255.4	654.1	412.09	90.78	22.03	0.496	−0.074
Huanxian	258.1	811.0	435.19	120.38	27.66	0.861	0.585
Jingyuan	135.4	416.8	234.59	62.36	26.58	0.778	0.457
Yuzhong	231.1	607.3	385.36	83.11	21.57	0.393	−0.208

**Figure 2:**
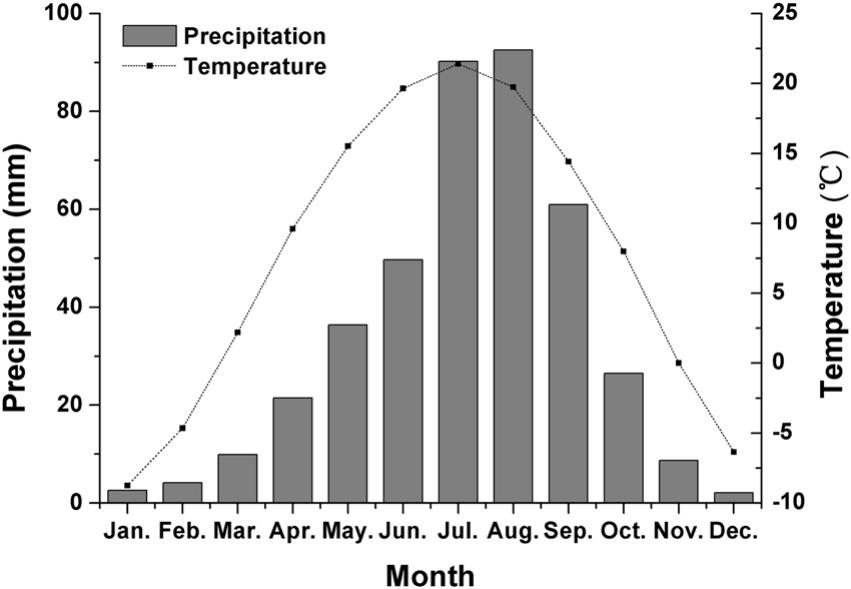
The average monthly precipitation and monthly mean temperature from 1958 to 2015.

The mean monthly precipitation and mean monthly air temperature data from 14 synoptic stations in the study area are shown in Fig. 2. Figure 2 shows that precipitation is mainly concentrated in July-September in the study area, with rain and high temperatures in the same period. The total amount of precipitation in these three months can account for more than 60% of the total annual precipitation; thus, this period is considered the rainy season in the study area.

## Precipitation trend analysis

### Serial correlation coefficient and trend-free prewhitening

In this study, formula (8) is used to calculate the autocorrelation coefficient of precipitation data at monthly, seasonal and annual scales. The results are shown in Table 2. According to formula (10), we conclude that lag-1 ∈ (−0.2748, 0.2398) (*n* = 58). Thus, the null hypothesis that the time series data show no autocorrelation was accepted. The data in Table 2 show that very few stations exhibit autocorrelation at the monthly, seasonal and annual time scales, and the TFPW method is used to analyze the precipitation time series with autocorrelation (dark area in the table). The values fall between −0.2748 and 0.2398; therefore, autocorrelation is eliminated from the original precipitation data. The next step is performing linear trend estimation, Spearman’s rho test and the M-K tests^29^.

**Table 2:** Lag-1 serial autocorrelation coefficient of precipitation at different time scales.

Station	Jan.	Feb.	Mar.	Apr.	May.	Jun.	Jul.	Aug.	Sep.	Oct.	Nov.	Dec.	Spring	Summer	Autumn	Winter	Annual
Wuqi	−0.028	−0.130	0.061	0.038	−0.070	−0.023	−0.050	0.048	0.096	−0.124	0.064	0.055	−0.004	−0.131	0.063	−0.021	−0.158
Suide	−0.094	−0.106	**0.038**	0.070	−0.126	0.016	−0.017	0.226	−0.058	−0.169	−0.099	−0.078	0.046	0.033	−0.079	−0.081	0.010
Hequ	0.095	−0.021	0.118	0.110	0.154	−0.112	−0.133	−0.015	0.055	−0.078	−0.260	−0.029	0.212	−0.150	0.069	0.168	−0.152
Xingxian	0.090	−0.162	−0.011	0.006	0.000	0.089	0.037	0.030	−0.410	−0.152	0.038	−0.104	0.050	−0.193	0.003	−0.166	−0.158
Wuzhai	0.224	−0.066	−0.061	0.098	0.012	**0.069**	0.062	−0.077	−0.165	−0.143	−0.073	−0.211	0.135	−0.013	−0.107	−0.108	−0.151
Youyu	0.010	−0.169	−0.004	0.103	−0.064	−0.267	0.059	−0.170	0.118	−0.134	−0.085	0.055	0.061	−0.254	0.089	−0.095	−0.253
Datong	0.085	−0.020	−0.047	−0.036	−0.070	0.109	−0.137	−0.270	0.044	0.153	−0.104	−0.043	−0.067	−0.119	0.086	−0.028	−0.244
Tongxin	−0.142	0.055	−0.029	−0.153	0.054	−0.060	0.133	−0.025	0.084	−0.149	0.003	−0.011	0.085	−0.063	0.115	0.216	−0.039
Haiyuan	−0.077	0.068	−0.108	−0.147	0.111	0.012	0.214	−0.027	−0.102	−0.159	0.149	0.013	0.128	0.002	**0.147**	0.081	−0.039
Guyuan	−0.056	0.174	−0.018	0.174	0.076	−0.064	0.168	**−0.056**	0.091	−0.079	−0.007	−0.036	0.051	−0.164	**−0.036**	**0.077**	−0.074
Xiji	0.075	0.101	−0.204	0.039	0.115	0.110	0.086	−0.087	0.043	−0.001	−0.035	−0.067	0.050	0.017	0.218	0.123	0.133
Huanxian	−0.068	0.019	0.047	0.197	−0.067	0.099	−0.045	−0.052	0.209	−0.016	−0.097	0.072	0.049	−0.179	**−0.055**	0.058	**−0.070**
Jingyuan	0.011	−0.081	−0.057	−0.139	0.112	−0.021	0.190	0.157	−0.103	−0.201	**0.036**	−0.038	0.074	0.169	−0.031	−0.019	0.071
Yuzhong	−0.088	0.152	−0.266	−0.183	−0.014	−0.005	0.178	0.003	−0.138	0.005	0.010	−0.142	−0.126	−0.084	0.082	**−0.001**	**−0.101**

Bold indicates that autocorrelations in the precipitation time series were corrected.

### Trend analysis of precipitation at different time scales

**Figure 3:**
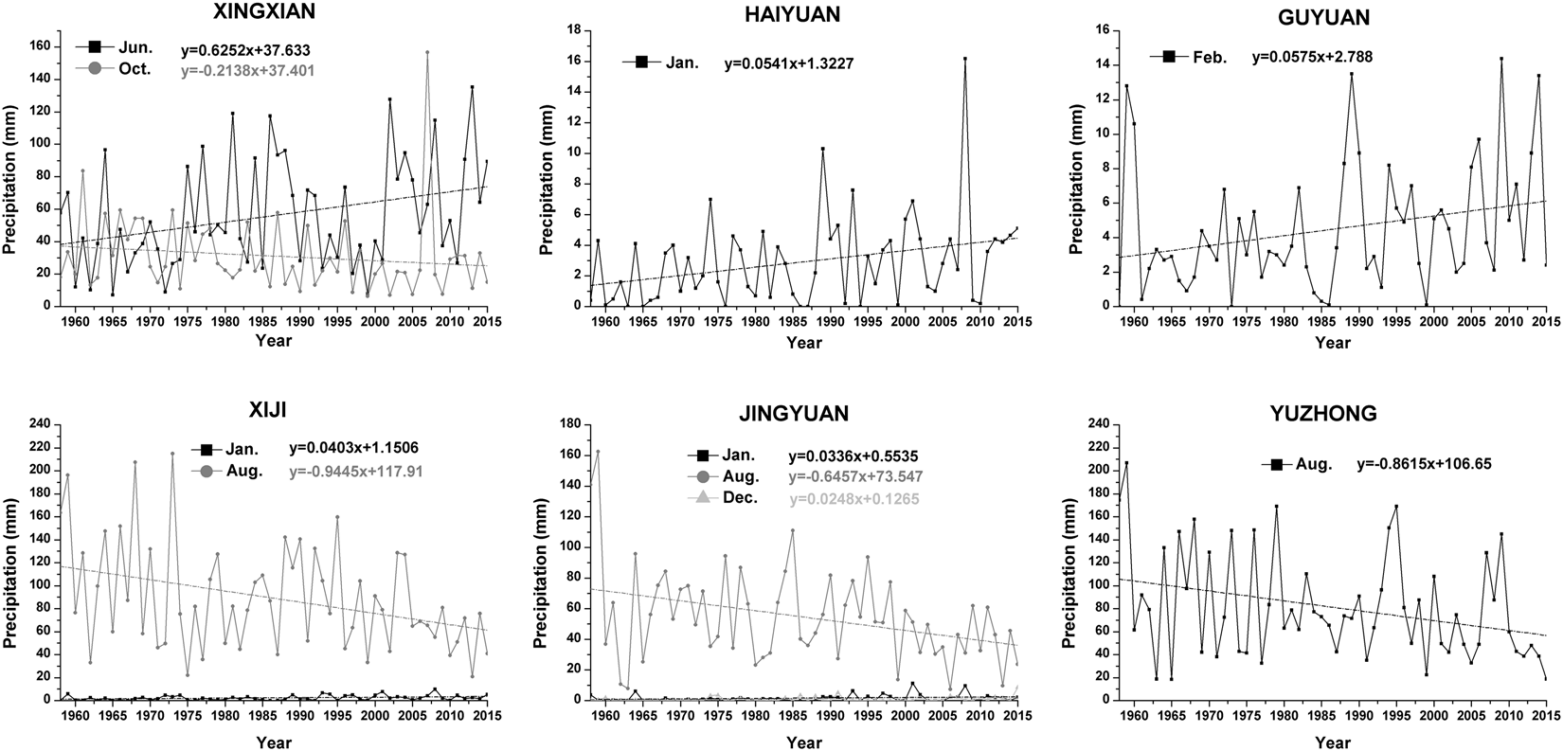
Variations in monthly precipitation at stations with significant trends during the study period.

Table 3 presents the trend test for monthly precipitation at the 14 synoptic stations in the study area from 1958 to 2015. The information in the table shows that the trend of precipitation at each station differs in different months. Overall, the number of synoptic stations that exhibited increasing trends or decreasing trends in January, May and December was relatively constant. Additionally, the majority of stations exhibited increasing trends in February and June. Only Hequ and Xingxian stations exhibited decreasing trends in February, and only Suide exhibited a decreasing trend in June. The stations exhibited downward trends in the remaining months. Notably, precipitation declined in July, August and September (rainy season) at most stations and in July and August at all stations. Among the stations, precipitation at Jingyuan, Yuzhong and Xiji stations in August decreased significantly by 0.978 mm/year, 0.646 mm/year and 0.862 mm/year, respectively. However, only a few months of precipitation trends were significant at the 95% level. All of the significant changes in the rainy season were decreasing trends, and the trends in other months were significant but weak increases. However, Xingxian exhibited a significant decreasing trend in October. The significant monthly precipitation trend at each synoptic station is shown in Fig. 3.

**Table 3:** Results of the statistical tests of monthly precipitation trends from 1958 to 2015.

Station	Test	Jan.	Feb.	Mar.	Apr.	May	Jun.	*Jul*.	*Aug*.	*Sep*.	Oct.	Nov.	Dec.
Wuqi	S	−0.310	0.886	−1.130	−1.290	−0.180	1.039	*−0.650*	*−1.390*	*−0.530*	−1.460	−0.360	1.054
	Z	−0.758	0.993	−1.207	−1.321	−0.195	0.959	*−0.517*	*−1.489*	*−0.543*	−1.442	−0.671	0.302
	b	−0.001	−0.007	−0.060	−0.182	−0.141	0.257	*−0.158*	*−0.590*	*−0.212*	−0.223	−0.014	0.015
Suide	S	−0.426	0.429	−0.970	−0.680	−0.570	−0.890	*−0.720*	*−1.760*	*0.313*	−1.260	−0.230	−0.020
	Z	−0.241	0.329	−0.906	−0.785	−0.631	−0.798	*−0.657*	*−1.771*	*0.282*	−1.201	−0.349	−0.852
	b	0.010	−0.012	−0.047	−0.126	−0.199	−0.114	*0.019*	*−0.854*	*0.078*	−0.284	0.138	−0.011
Hequ	S	−0.200	−0.280	−1.710	−0.530	1.917	1.977	*−2.070*	*−0.980*	*−0.130*	−1.350	0.307	−0.270
	Z	−0.503	−0.577	−1.744	−0.617	1.603	1.838	*−2.026*	*−0.979*	*−0.074*	−1.389	0.027	−0.711
	b	−0.028	−0.010	−0.071	−0.115	0.189	0.277	*−0.807*	*−0.697*	*−0.164*	−0.119	0.092	−0.016
Xingxian	S	−0.030	−0.100	−1.480	0.102	1.709	**2.214**	*−1.390*	*−0.810*	*0.203*	**−2.080**	0.477	−0.100
	Z	−0.087	−0.195	−1.529	−0.067	1.563	**2.026**	*−1.409*	*−0.771*	*0.382*	**−2.160**	0.268	−0.215
	b	0.002	−0.022	−0.082	−0.018	0.244	**0.625**	*−0.495*	*−0.720*	*−0.060*	**−0.214**	0.078	0.022
Wuzhai	S	0.616	1.690	−1.550	0.931	2.016	1.406	*−1.080*	*−0.140*	*0.792*	0.085	0.102	−0.390
	Z	0.369	1.570	−1.576	0.852	1.932	1.228	*−0.946*	*−0.020*	*0.906*	0.107	0.007	−0.476
	b	0.007	0.065	−0.036	0.034	0.223	0.268	*−0.642*	*−0.248*	*0.243*	0.020	0.104	0.009
Youyu	S	−0.480	0.717	−1.100	0.689	1.563	0.488	*−1.380*	*−1.340*	*0.420*	−0.170	0.146	0.941
	Z	−0.698	0.617	−1.127	0.483	1.456	0.423	*−1.489*	*−1.328*	*0.342*	−0.255	0.000	0.792
	b	−0.015	0.011	−0.059	0.065	0.232	0.246	*−0.431*	*−0.634*	*0.090*	−0.011	0.084	0.007
Datong	S	−0.540	0.460	−0.920	0.675	0.906	1.862	*−1.350*	*−1.210*	*1.125*	−0.250	0.312	−0.490
	Z	−1.033	0.356	−1.020	0.483	0.979	1.851	*−1.516*	*−1.207*	*1.033*	−0.107	0.000	−0.235
	b	−0.024	0.003	−0.045	0.043	0.105	0.302	*−0.371*	*−0.472*	*0.293*	−0.029	0.070	0.003
Tongxin	S	0.373	0.358	−0.870	−1.240	−0.840	1.476	*−0.390*	*−1.150*	*−0.560*	−1.180	−1.450	1.175
	Z	−0.034	0.127	−1.026	−1.389	−1.060	1.597	*−0.396*	*−1.093*	*−0.597*	−1.248	−1.737	−0.161
	b	0.012	−0.009	−0.040	−0.096	−0.121	0.297	*−0.073*	*−0.556*	*−0.065*	−0.122	−0.085	0.008
Haiyuan	S	**2.788**	0.080	0.157	−0.770	−0.120	0.379	*0.083*	*−0.914*	*−0.110*	−0.660	−0.960	1.774
	Z	**2.489**	0.631	0.134	−0.751	−0.134	0.302	*0.402*	*−1.825*	*−0.101*	−0.704	−1.020	1.261
	b	**0.054**	0.147	−0.033	−0.010	−0.030	0.179	*−0.092*	*−1.914*	*−0.028*	−0.155	−0.095	0.040
Guyuan	S	1.031	**2.323**	1.319	−0.370	−1.070	0.115	*−1.580*	*−0.740*	*−0.860*	0.003	−1.090	1.587
	Z	0.892	**2.200**	1.261	−0.396	−0.966	0.000	*−1.536*	*−0.879*	*−0.751*	0.060	−1.281	1.221
	b	0.039	**0.058**	0.072	0.016	−0.211	0.089	*−0.543*	*−0.461*	*−0.297*	−0.073	−0.046	0.029
Xiji	S	**2.643**	0.970	0.087	−0.140	−0.430	1.100	*−1.110*	***−2.490***	*−0.600*	−0.160	−0.460	0.957
	Z	**2.368**	0.979	0.0738	−0.101	−0.617	1.060	*−1.060*	***−2.375***	*−0.530*	−0.188	−0.530	0.503
	b	**0.040**	0.016	−0.022	0.029	−0.075	0.410	*−0.324*	***−0.978***	*−0.256*	−0.089	−0.016	0.010
Huanxian	S	0.673	0.584	−1.220	−0.310	−0.770	1.676	*−0.500*	*−1.040*	*−0.690*	−0.760	−1.000	0.983
	Z	0.322	0.456	−1.321	−0.416	−0.711	1.617	*−0.570*	*−1.040*	*−0.724*	−0.691	−1.194	0.107
	b	0.024	0.0001	−0.062	−0.006	−0.062	0.405	*−0.128*	*−0.741*	*−0.208*	−0.216	−0.023	0.017
Jingyuan	S	**2.643**	0.500	−0.670	−0.900	0.012	1.098	*−0.820*	***−2.380***	*−0.790*	−0.630	−0.520	**4.467**
	Z	**1.925**	0.127	−0.939	−0.865	0.054	1.067	*−0.698*	***−2.462***	*−0.758*	−0.704	−0.349	**2.294**
	b	**0.034**	0.0004	−0.038	−0.080	0.015	0.200	*−0.244*	***−0.646***	*−0.098*	−0.080	−0.038	**0.025**
Yuzhong	S	0.935	1.865	0.056	−0.38	0.174	1.041	*−0.990*	***−2.150***	*−0.980*	−0.350	−0.190	−0.150
	Z	0.617	1.617	−0.054	−0.389	0.188	1.020	*−0.926*	***−2.213***	*−0.939*	−0.416	−0.127	−0.409
	b	0.014	0.048	−0.015	−0.101	0.052	0.265	*−0.232*	***−0.862***	*−0.219*	−0.094	−0.052	−0.007

Cells in italic indicate the rainy season. Z: M-K trend test; S: Spearman’s rho test; and b (mm/a): slope of linear regression. Bold type represents trends identified by 2 statistical methods and trends that are statistically significant at the 5% level.

The results of the trend analysis of seasonal and annual precipitation at each synoptic station are shown in Table 4. According to the information in the table, the variations in precipitation in different seasons differ at different synoptic stations. Notably, two trends can be observed in the spring, autumn and winter at each synoptic station, and the only summer trend is a decreasing trend. In spring, the number of synoptic stations that exhibited each type of trend was relatively equal, while more stations exhibited decreasing trends in autumn, and more stations exhibited increasing trends in winter. All significant trends occurred in winter (Haiyuan station, Guyuan station and Xiji station) and were increasing trends with rates of 0.082 mm/year (Haiyuan), 0.071 mm/year (Guyuan) and 0.058 mm/year (Xiji). The annual precipitation trends indicate that annual precipitation at almost all stations exhibited a decreasing trend (only Wuzhai station exhibited a non-significant increase trend). Although there no significant increasing or decreasing trend was observed, the results suggest that annual precipitation is associated with a decreasing trend in the water-wind erosion crisscross region on the Loess Plateau.

**Table 4:** Results of the statistical tests of seasonal and annual precipitation in the study period.

Station	Test	Spring	Summer	Autumn	Winter	Annual
Wuqi	S	−1.390	−0.840	−0.780	0.436	−1.270
	Z	−1.355	−0.885	−0.711	0.443	−1.221
	b	−0.384	−0.491	−0.449	−0.009	−1.316
Suide	S	−0.920	−1.650	−0.060	0.156	−1.920
	Z	−0.745	−1.690	−0.094	0.141	−2.073
	b	−0.372	−0.949	−0.068	−0.012	−1.402
Hequ	S	0.603	−1.280	−0.120	−1.440	−1.100
	Z	0.530	−1.382	−0.114	−1.482	−1.067
	b	0.003	−1.226	−0.192	−0.054	−1.470
Xingxian	S	0.644	−1.050	−0.200	−0.210	−0.470
	Z	0.738	−1.093	−0.221	−0.288	−0.389
	b	0.145	−0.590	−0.196	0.004	−0.640
Wuzhai	S	1.351	−0.670	1.460	1.853	0.162
	Z	1.295	−0.678	1.630	1.764	0.114
	b	0.220	−0.665	0.367	0.084	0.003
Youyu	S	1.470	−1.340	0.561	0.458	−0.290
	Z	1.114	−1.395	0.584	0.496	−0.369
	b	0.238	−0.818	0.162	−0.001	−0.415
Datong	S	0.596	−1.410	1.045	−0.870	−0.010
	Z	0.657	−1.550	0.959	−0.912	0.134
	b	0.104	−0.541	0.334	−0.019	−0.122
Tongxin	S	−1.420	−0.450	−1.140	−0.060	−1.100
	Z	−1.456	−0.470	−1.348	−0.040	−1.160
	b	−0.258	−0.331	−0.272	−0.004	−0.849
Haiyuan	S	−0.200	−0.620	−0.640	**2.156**	−1.150
	Z	−0.322	−0.617	−0.590	**2.033**	−1.187
	b	−0.073	−0.827	−0.277	**0.082**	−1.069
Guyuan	S	−0.700	−1.010	−0.870	**2.274**	−1.580
	Z	−0.778	−1.053	−0.785	**2.220**	−1.556
	b	−0.123	−0.858	−0.379	**0.071**	−1.271
Xiji	S	−0.300	−1.680	−0.770	**2.107**	−1.820
	Z	−0.537	−1.751	−0.711	**2.093**	−1.818
	b	−0.068	−0.891	−0.358	**0.058**	−1.250
Huanxian	S	−0.590	−0.580	−0.800	1.087	−1.020
	Z	−0.637	−0.724	−0.906	1.040	−1.033
	b	−0.130	−0.155	−0.378	0.027	−0.999
Jingyuan	S	−0.240	−1.250	−1.250	1.314	−1.860
	Z	−0.228	−1.281	−1.214	1.207	−1.878
	b	−0.104	−0.689	−0.227	0.044	−0.961
Yuzhong	S	0.149	−1.270	−1.350	1.521	−1.710
	Z	0.215	−1.308	−1.254	1.536	−1.597
	b	−0.063	−0.829	−0.365	0.041	−1.202

Z: M-K trend test; S: Spearman’s rho test; and b (mm/a): slope of linear regression. Bold type represents trends identified by 2 statistical methods and trends that are statistically significant at the 5% level.

### Trend analysis of erosive rainfall

**Figure 4:**
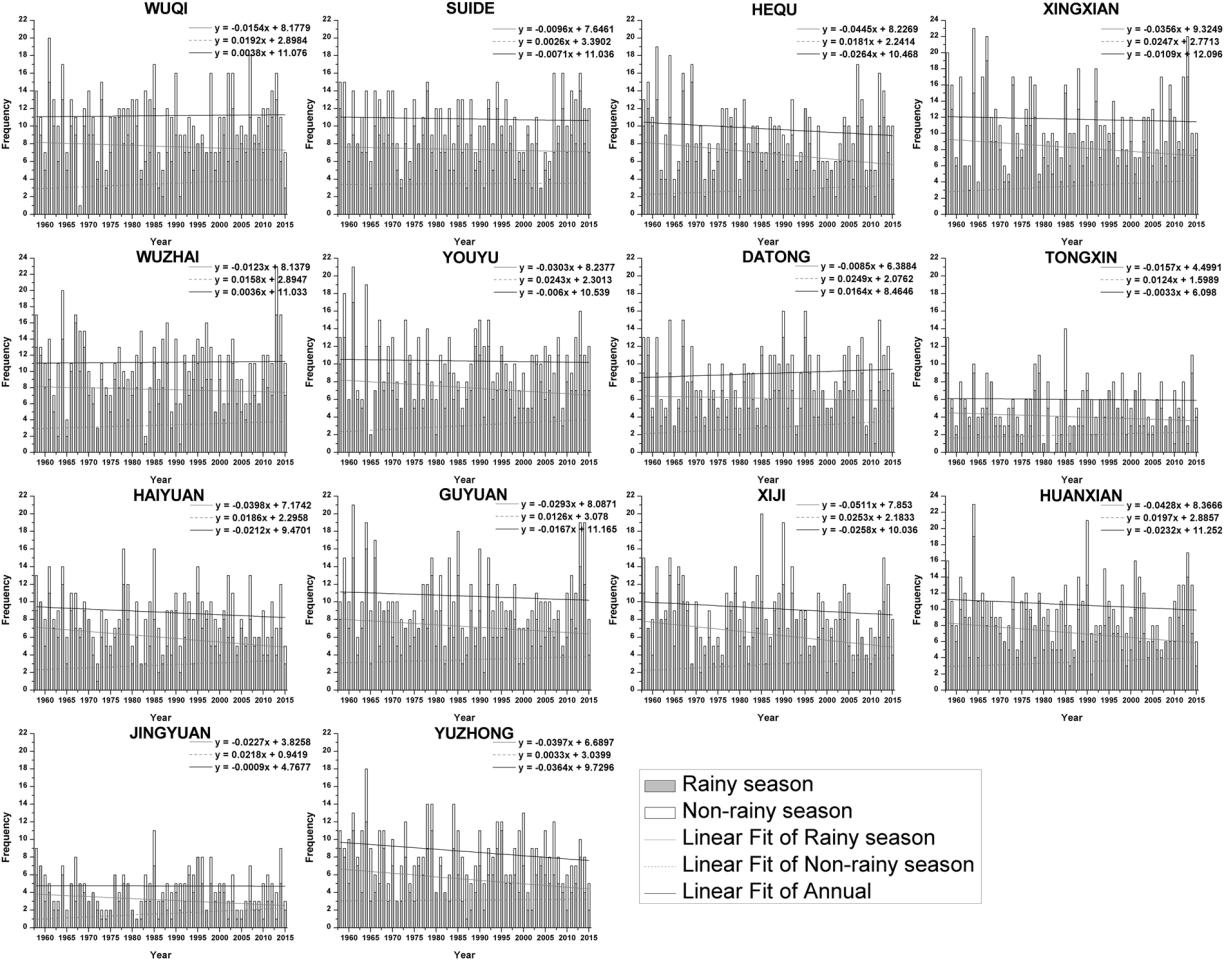
Frequency and trend of erosive rainfall in rainy season and non-rainy season.

**Figure 5:**
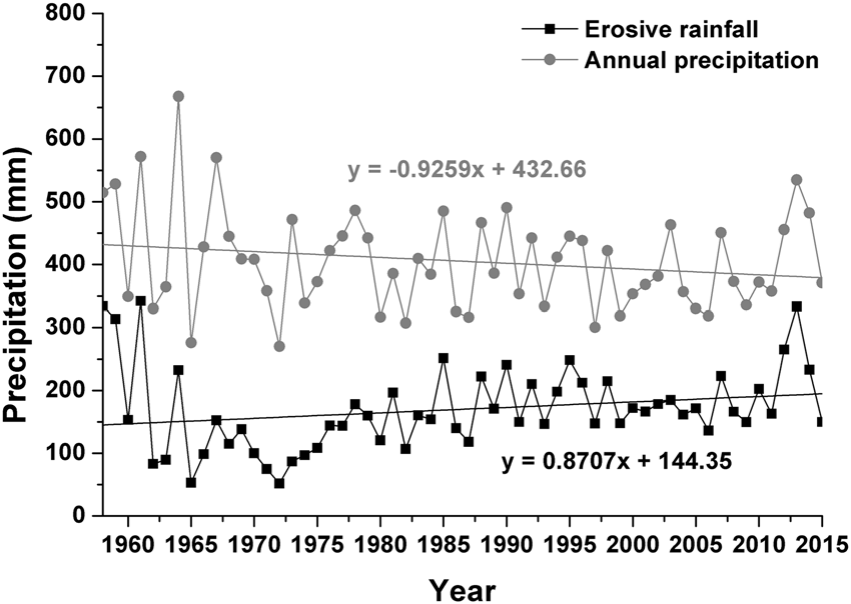
Trend of annual precipitation and erosive precipitation in the study area.

Daily rainfall greater than 12 mm has been used as the standard for describing erosive rainfall on the Loess Plateau^24–26^. In the study area, the erosive rainfall occurred in the rainy season of each synoptic station was more frequent than non-rainy season (Fig. 4). The change trend of frequency of erosive rainfall (rainy season, non-rainy season and annual) was analyzed by linear trend estimation, and the results showed that there were differences in the frequency of occurrence of erosive rainfall at different timescales, for specific performance: it presented a decrease trend in rainy season and a increase trend in non-rainy season, however, most of synoptic stations also showed a decrease trend but the trend was weak. In terms of precipitation depth, the relationship between average annual erosive rainfall (*x*) and annual precipitation (*y*) in the study area were analyzed, and the result showed that there was a not quite obvious linear relationship between these two parameters from 1958 to 2015 (*y* = 0.568*x*–60.01, R = 0.684, P < 0.001). However, the average annual precipitation in the whole study area showed a decreasing trend but the erosive rainfall was gradually increased (Fig. 5).

The frequency of erosive rainfall in the rainy season and the annual scale was reduced but the erosive precipitation depth in the corresponding period was increased, this indicated the erosive force of single rainfall in the study area has been enhanced that during the study period.

### SPI analysis

Over a timescale of 1–6 months, the SPI can reflect agricultural drought conditions, and over a longer timescale (6 months or longer), it can be used to research hydrological drought in groundwater, rivers, and reservoirs^3, 6, 7^.

**Figure 6:**
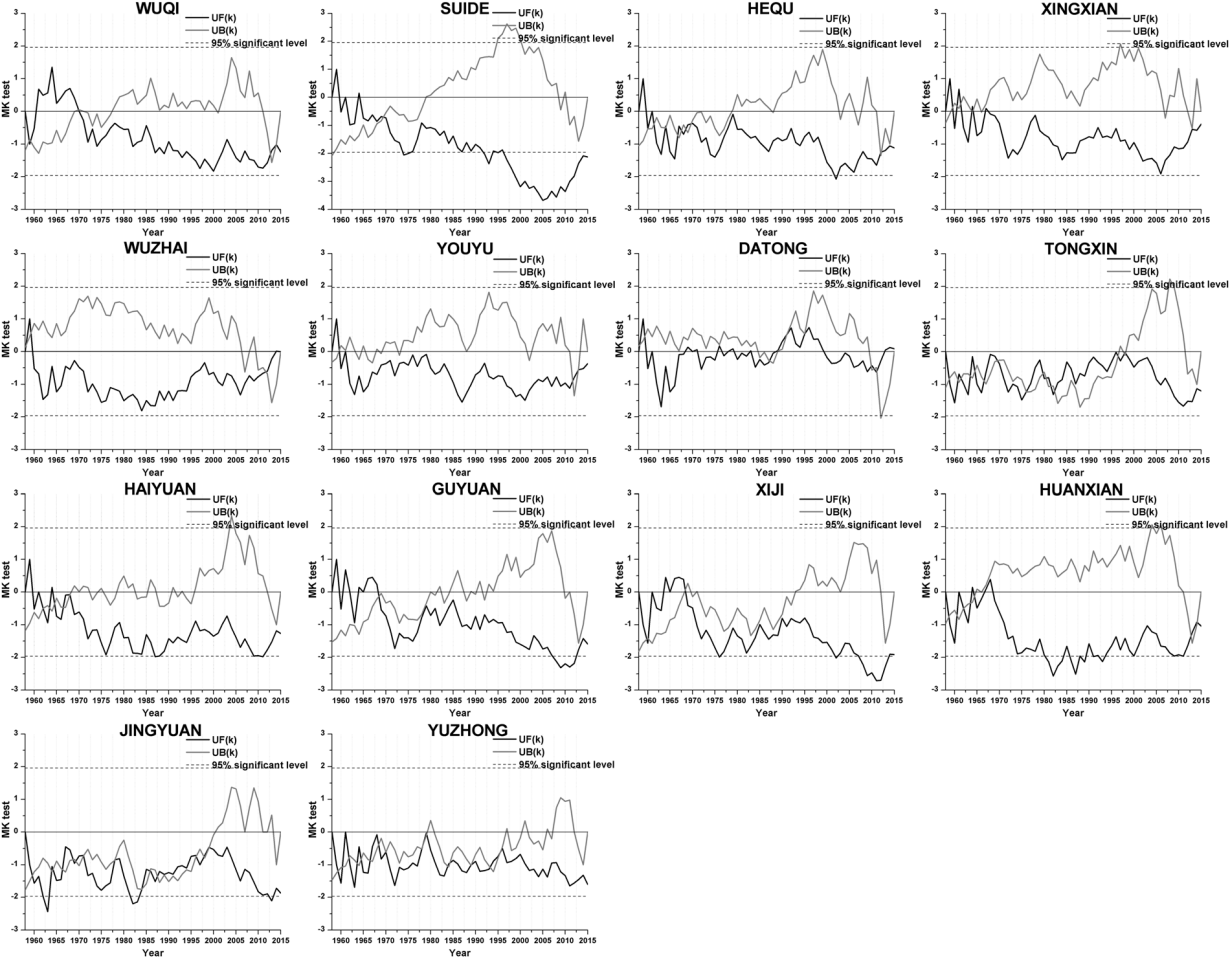
M-K trend test of annual SPI.

In the SPI trend analyses at seasonal and annual scales, the SPI-3 (3-month scale) values in May, August, November and February represent spring, summer, autumn and winter, respectively, and SP-12 (12-month scale) values in December represent the whole year. The trend analyses of seasonal and annual SPI (Table 5) showed that the seasonal SPI differed at each station. Specifically, in the spring, summer and autumn, the SPI exhibited a decreasing trend, while the SPI generally increased in winter. Thus, spring, summer, autumn are becoming more arid, and the winter is becoming more humid. Annual SPI exhibited a decreasing trend at all stations except Wuzhai and Datong. Although the trends are not significant, the SPI values in the study area are trending toward drought conditions, and the occurrence of drought will likely continue to increase. The M-K abrupt analysis of annual SPI at each station (Fig. 6) showed that the SPI at Wuzhai station decreased from 1960 to 2012. The SPI then abruptly increased and continued to increase until 2015. The annual SPI in Datong fluctuated, including increases from 1990–2000 and after 2013. The UF_*k*_ curves of other stations are always below the y = 0 line, except those of Wuzhai and Datong. Thus, the annual SPI trend is continuously and stably decreasing at most stations, but the annual SPI trends at Wuzhai and Datong are unstably increasing.

**Table 5:** Results of the statistical tests of seasonal and annual SPI in the study period.

Station	Test	Spring	Summer	Autumn	Winter	Annual
Wuqi	S	−1.351	−0.829	−0.738	0.826	−1.234
	Z	−1.382	−0.879	−0.718	0.382	−1.214
Suide	S	−0.892	−1.582	−0.035	0.242	−1.924
	Z	−0.771	−1.717	−0.067	0.094	−2.100
Hequ	S	0.691	−1.259	−0.058	−1.077	−1.091
	Z	0.557	−1.362	−0.087	−1.409	−1.080
Xingxian	S	0.688	−1.040	−0.200	−0.245	−0.418
	Z	0.711	−1.093	−0.255	−0.268	−0.369
Wuzhai	S	1.358	−0.675	1.478	2.029	0.136
	Z	1.254	−0.691	1.597	1.766	0.054
Youyu	S	1.537	−1.311	0.606	1.024	−0.278
	Z	1.134	−1.382	0.637	0.651	−0.342
Datong	S	0.742	−1.389	1.110	−0.252	0.037
	Z	0.684	−1.536	0.953	−0.751	0.121
Tongxin	S	−1.333	−0.394	−1.118	0.512	−1.039
	Z	−1.436	−0.449	−1.355	0.007	−1.147
Haiyuan	S	−0.131	−0.577	−0.595	**2.484**	−1.184
	Z	−0.282	−0.584	−0.644	**2.046**	−1.221
Guyuan	S	−0.619	−1.001	−0.781	**3.600**	−1.574
	Z	−0.765	−1.046	−0.704	**3.173**	−1.550
Xiji	S	−0.293	−1.668	−0.732	**2.545**	−1.819
	Z	−0.543	−1.744	−0.731	**2.093**	−1.858
Huanxian	S	−0.538	−0.570	−0.776	1.582	−0.980
	Z	−0.631	−0.718	−0.919	1.120	−0.999
Jingyuan	S	−0.138	−1.163	−1.129	2.054	−1.842
	Z	−0.235	−1.275	−1.160	1.362	−1.818
Yuzhong	S	0.202	−1.263	−1.267	**2.453**	−1.593
	Z	0.221	−1.321	−1.221	**1.872**	−1.529

Z: M-K trend test; S: Spearman’s rho test; and b (mm/a): slope of linear regression. Bold type represents trends identified by 2 statistical methods and trends that are statistically significant at the 5% level.

**Figure 7:**
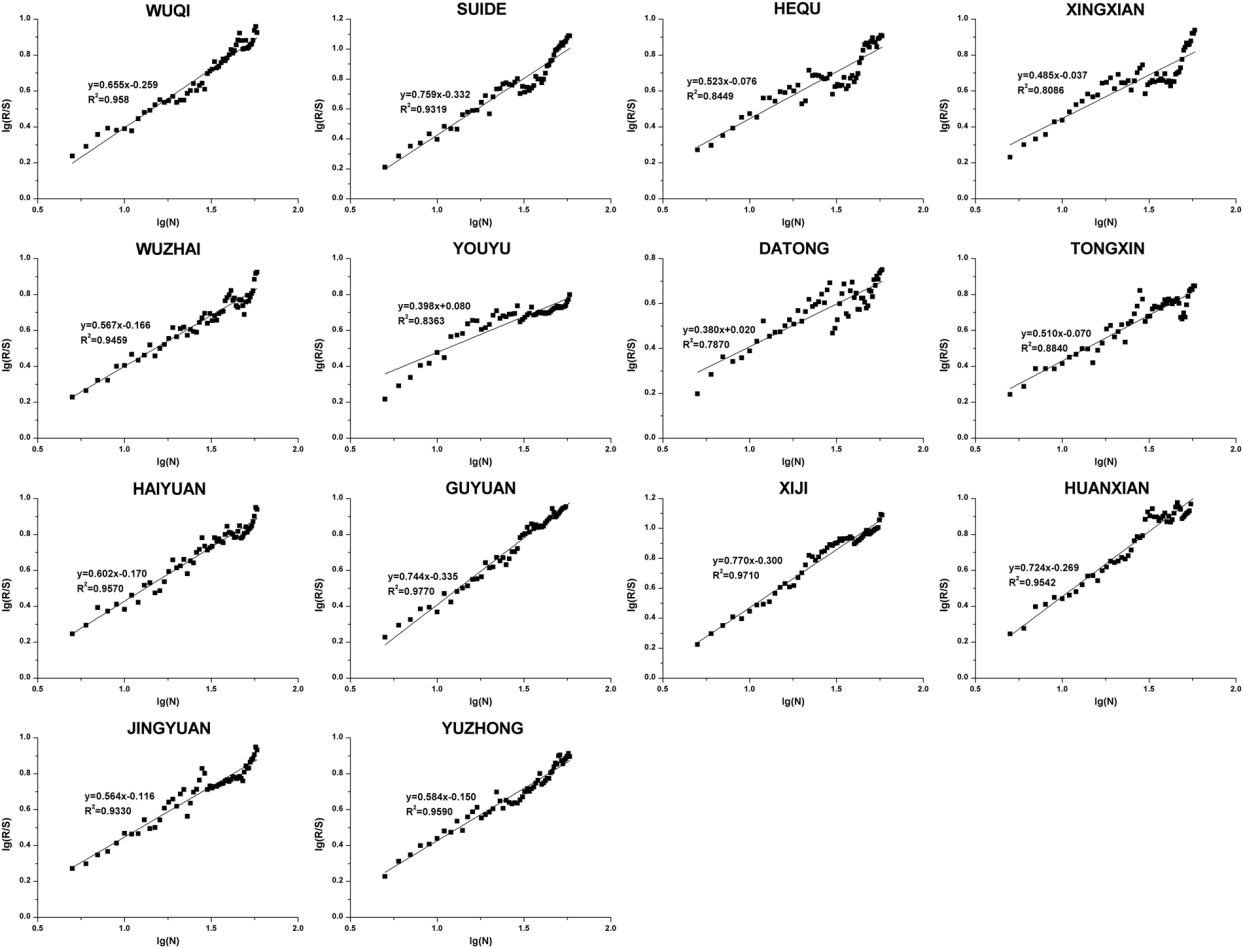
The rescaled range analysis of SPI-12 at different stations.

The M-K trend test and Spearman’s rho test were mainly used to analyze the current trends of time series, whereas the R/S analysis was mainly performed to evaluate the long-term correlation of time series^30, 31^. The combination of the three methods can not only clarify the current trends of time series but also predict future trends to a certain extent. As illustrated by the Hurst index values of the annual SPI at each station (Fig. 7), only Xingxian (H = 0.485), Youyu (H = 0.398) and Datong (H = 0.380) exhibit trends that are predicted to change, and other stations will likely maintain current trends. The annual SPI at Xingxian and Youyu may increase, which is different from the current trend, while the annual SPI at Datong is likely to decrease in the future. Based on Figs 6 and 7, although the annual SPIs of the above three stations may exhibit different trends in the future, most of the stations still exhibit stable and sustained downward trends. Therefore, the degree of drought will likely continue to increase in the future.

**Figure 8:**
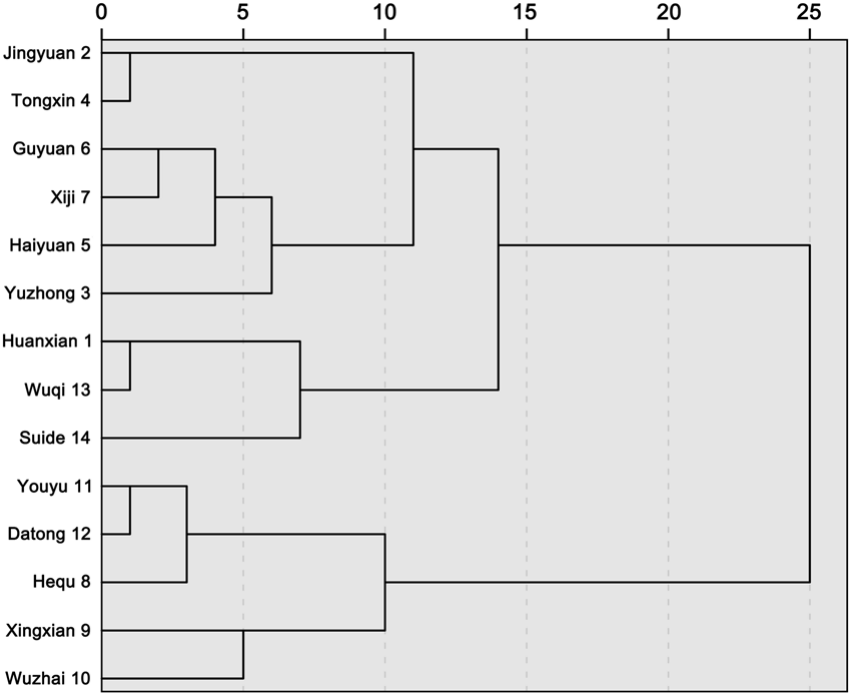
Hierarchical diagram of the cluster analysis of monthly precipitation.

**Figure 9:**
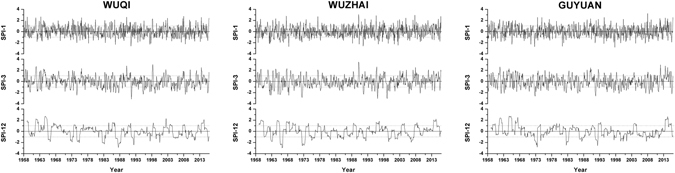
Different time scales of SPI (SPI-1, SPI-3, SPI-12) at 3 representative synoptic stations from 1958 to 2015.

SPI is calculated based on monthly precipitation. In this study, a cluster analysis based on the Ward method was performed using monthly precipitation data collected at 14 synoptic stations from 1958–2015. According to the results of the cluster analysis (Fig. 8), three stations from different cluster types but with similar values of annual precipitation were chosen to analyze SPI-1 (1-month scale), SPI-3 and SPI-12 trends: Wuqi (470.25 mm), Wuzhai (474.15 mm) and Guyuan (455.54 mm). As shown in Fig. 9, both monthly scale SPI-1 and seasonal scale SPI-3 are sensitive to short-term precipitation changes. Additionally, these values fluctuate considerably and fully reflected the short-term characteristics of the study area with respect to impermanent droughts and frequent variations. Moreover, the annual SPI-12 shows that the periodicity of drought and flood fluctuation is more obvious and stable.

**Figure 10:**
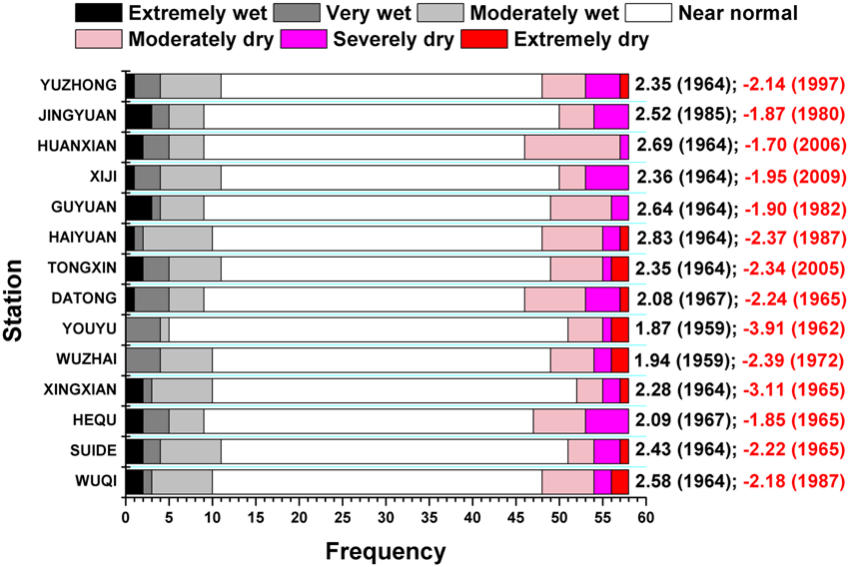
Frequencies of dry and wet phenomena. (The black and red numbers on the right represent the maximum and minimum SPI-12, respectively, and the corresponding year is given in parentheses).

The value of SPI-12 in December can be used to determine the dry and wet conditions in the subsequent year. The frequencies of dry, wet and normal years at each station are shown in Fig. 10. The figure shows that the frequencies of wet and dry years differ at different synoptic stations, and normal years are most frequent at all stations. According to the analysis of SPI-12, the frequency of wet years is highest (11 years) at Suide, Tongxin, Xiji and Yuzhong stations, and nine of the 14 stations (Yuzhong, Xingxian, Xiji, Wuqi, Tongxin, Suide, Huanxian, Haiyuan and Guyuan) were the wettest in 1964. From 1958 to 2015, the frequency of drought was highest (11 years) at Datong and Huanxian and the lowest at Xingxian (6 years). During the study period, the most severe drought in the study area was at Yuli station in 1962, and the wettest year was observed at Haiyuan station in 1964.

## Discussion and Conclusions

Precipitation is closely related to hydraulic erosion and drought, but drought often causes severe wind erosion. Compared with other regions of the Loess Plateau, precipitation plays a more important role in the water-wind erosion crisscross region. Additionally, precipitation is the main form of soil water recharge on the Loess Plateau, and soil water is the main limiting factor of vegetation restoration. Fully understanding the characteristics of precipitation and dry/wet conditions in the water-wind erosion crisscross region can improve the management of regional water resources, the water use efficiency, the efficiency of vegetation restoration, and the ecological environment in the region. To understand the characteristics of precipitation and dry/wet conditions, this study analyzed these characteristics and variations in the SPI at different time scales based on observations from 14 synoptic stations in the study area. The analysis included trend-free prewhitening, linear trend estimation, Spearman’s rho test, the M-K trend test, the M-K abrupt change test and rescaled range analysis.

The variations in precipitation differed at different time scales. Notably, monthly precipitation in different months varied at different stations. These differences may be related to the fractured topography of the study area^32^, but rainy season precipitation, especially that in July and August, generally exhibited a decreasing trend. Winter precipitation at most stations in the study area displayed an increasing trend and most stations exhibited decreasing trends in the spring, summer autumn. Specifically, the annual precipitation at Wuzhai station began to shift to a non-significant increasing trend, and the rest of the stations exhibited decreasing trends. The water-wind erosion crisscross region on the Loess Plateau is located in the monsoon region of China. Previous studies showed that the monsoon climate in China is influenced by the East Asian summer monsoon^33, 34^, and the East Asian summer monsoon is strongest in July and then begins to weaken until it ends^35^. Recent studies have shown that the East Asian summer monsoon is weakening^36, 37^, which may be one of the main reasons for the decreases in precipitation in July and August and in summer and autumn. Additionally, the winter monsoon index is negatively correlated with precipitation in Northwest China. The weakening of the winter monsoon^38^, which reduces the transport of dry and cold air to the study area, may increase precipitation to a certain extent.

The SPI is calculated from monthly precipitation. Therefore, the results of the trend analysis of SPI series were similar to the results of the precipitation series analysis at different scales. The frequencies of wet and dry years vary from station to station, but the frequency of normal years is the highest at each station. Seasonal SPI exhibited a decreasing trend in spring, summer and autumn at most stations and an increasing trend in winter. The annual SPI at most stations in the study area displayed a stable and sustained downward trend, which suggests that the water-wind erosion crisscross region of the Loess Plateau is currently associated with a drought trend, and the drought degree will likely continue to increase.

Analyses of precipitation data and SPI series can improve the efficiency of water resource use for hydroelectric and agricultural production. Although the study area was always in the trend of drought but the drier-and-hotter trend has been slightly restrained to varying levels since the implementation of the “Grain for Green” policy^39, 40^. The trend of drought may cause more wind erosion and the increase in erosive precipitation depth enhanced the hydraulic erosion force, but the erosion status of the study area has been effectively inhibited by increasing the vegetation coverage since the “Grain for Green” policy^41, 42^, it can be seen that vegetation restoration is an effective ecological means for improving the environmental conditions of study area to a certain extent, at the same time, vegetation cover can also reduce soil erosion^43, 44^. Better precipitation and temperature conditions are also more suitable for the progress of vegetation restoration. The research areas studied as part of the “China National Scientific and Technical Innovation Research Project for 13th Five Year Plan” include the water-wind erosion crisscross region of the Loess Plateau. Vegetation restoration technology and the relationship between the Normalized Difference Vegetation Index^45^ and climate change should be studied in this area to further improve the regional environment.

## Material and methods

### Study area and data

**Figure 11:**
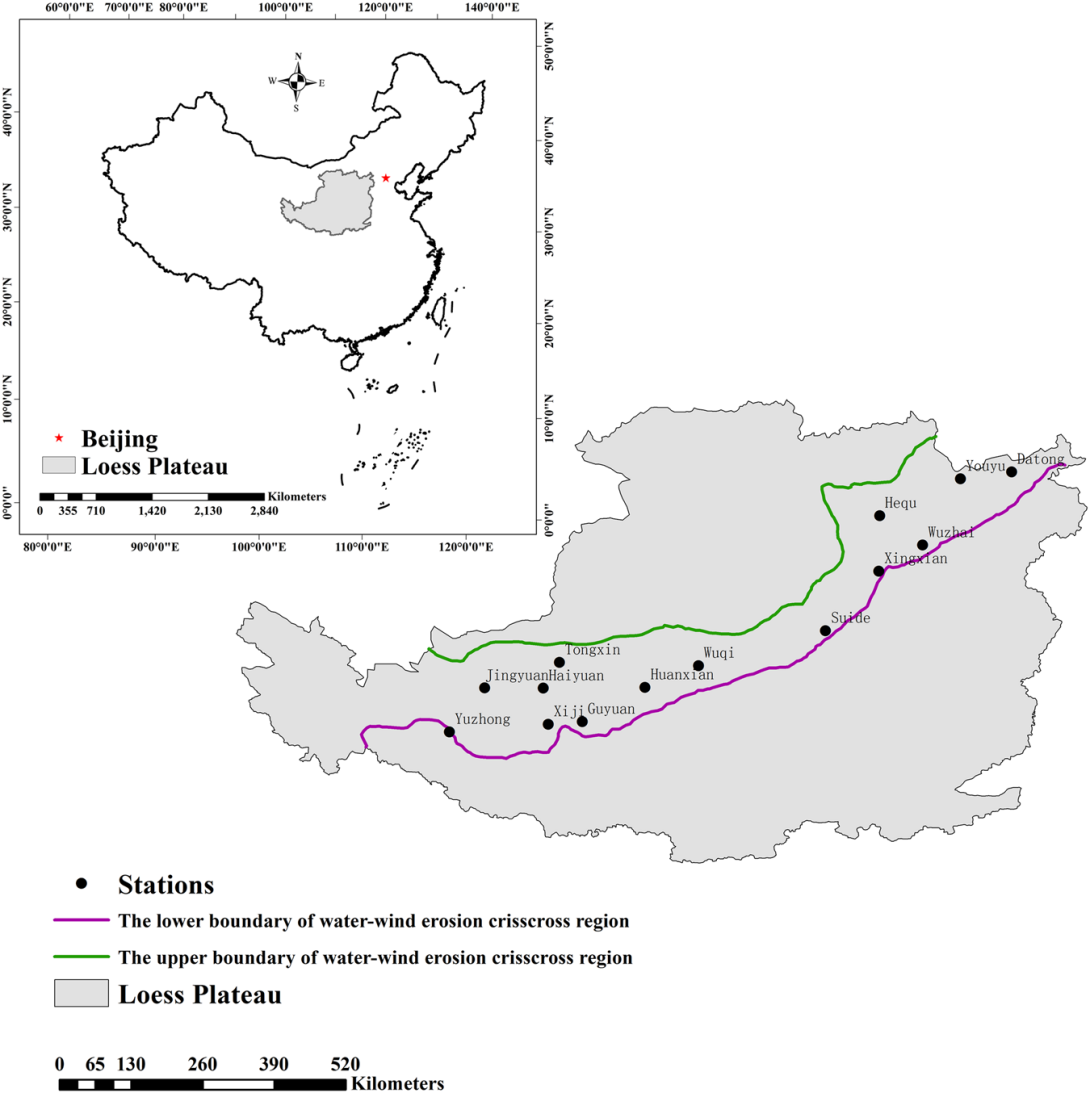
Study area and spatial distribution of the 14 synoptic stations. (The two maps were created using ArcGis 10.2 and then combined together using Adobe Photoshop CS6. ArcGIS 10.2: http://www.esri.com/apps/products/download/index.cfm?fuseaction=download.all#ArcGIS_Desktop; Adobe Photoshop CS6: http://www.adobe.com/cn/products/cs6/photoshop.html).

The water-wind erosion crisscross region on the Loess Plateau (Fig. 11) is located in the transition region between the hill and gully loess region and the Maowusu Desert (35°25′N–40°38′N, 103°00′E–13°53′E). Climate change is intense in this area, and vegetation is sparse. The northern part of the water-wind erosion crisscross region on the Loess Plateau is the wind erosion area, and the southern portion is the water erosion area.

The meteorological data used in this study were obtained from the China Meteorological Data Network (http://data.cma.cn/). To ensure synchronization between meteorological data and the long-term series of observations collected at each synoptic station, stations with missing data were excluded. A total of 14 representative stations were selected for this study (Table 6). Additionally, precipitation data from 1958 to 2015 were used.

**Table 6:** Geographical descriptions of the synoptic stations used in this study.

No.	Station	Longitude (E)	Latitude (N)	Elevation (m a.s.l.)	Province (Autonomous region)
53738	Wuqi	108.10	36.55	1331.1	Shaanxi
53754	Suide	110.13	37.30	930.7	Shaanxi
53564	Hequ	111.09	39.23	862.5	Shanxi
53664	Xingxian	111.08	38.28	1013.6	Shanxi
53663	Wuzhai	111.49	38.55	1402	Shanxi
53478	Youyu	112.27	40.00	1346.7	Shanxi
53487	Datong	113.25	40.05	1053.6	Shanxi
53810	Tongxin	105.54	36.58	1340.7	Ningxia
53806	Haiyuan	105.39	36.34	1854.8	Ningxia
53817	Guyuan	106.16	36.00	1754.2	Ningxia
53903	Xiji	105.43	35.58	1917.9	Ningxia
53821	Huanxian	107.18	36.34	1256.8	Gansu
52895	Jingyuan	104.41	36.34	1399.4	Gansu
52983	Yuzhong	104.09	35.52	1875.6	Gansu

## Study Methods

### Standardized precipitation index

The SPI is a drought index recommended by the WMO, and it has been used in many studies^4, 46^. The calculation steps are as follows:

1 $${\rm{SPI}}=S\frac{t-({c}_{2}t+{c}_{1})t+{c}_{0}}{[({d}_{3}t+{d}_{2})t+{d}_{1}]t+1}$$

where $t=\sqrt{\mathrm{ln}\,\frac{1}{G{(x)}^{2}}}$, *G*(*x*), the probability distribution of precipitation related to the gamma function, is calculated as follows:

2 $$G(x)=\frac{1}{{\alpha }^{\beta }\Gamma {(\beta )}_{0}}{\int }_{0}^{x}{x}^{\beta -1}{e}^{-x/\alpha }dx,\,x > 0$$

where *x* is the precipitation sequence. *S* is the probability density distribution, namely, *S* = 1 when *G*(*x*) > 0.5 and *S* = −1 when *G*(*x*) ≤ 0.5. Γ(*β*) is the gamma function of *β*, where *c*_0_ = 2.515517, *c*_1_ = 0.802853, *c*_2_ = 0.010328, *d*_1_=1.432788, *d*_2_ = 0.189269, and *d*_3_ = 0.001308. *α* and *β* are scale and shape parameters, respectively, and can be obtained as follows.

3 $$\alpha =\frac{({w}_{0}-2{w}_{1})\beta }{\Gamma (1+\frac{1}{\beta })\Gamma (1-\frac{1}{\beta })}$$

4 $$\beta =\frac{2{w}_{1}-{w}_{0}}{(6{w}_{1}-{w}_{0}-6{w}_{2})}$$

5 $${w}_{s}=\frac{1}{n}\sum _{l=1}^{n}{(1-\frac{l-0.35}{n})}^{s}{X}_{l}$$

In Eq. (5), *w*_*s*_ is the probability weighted moment, where *s* = 0, 1, 2, and *l* is the ordinal number of the precipitation sequence *x* in ascending order.

The tentative range of the SPI and the drought-wetness grade classification criteria are shown in Table 7.

**Table 7:** Categories of drought grade based on SPI.

SPI	Classification
≥2.0	Extremely wet
1.5 ~ 1.99	Very wet
1.0 ~ 1.49	Moderately wet
−0.99 ~ 0.99	Near normal
−1.0 ~ −1.49	Moderately dry
−1.5 ~ −1.99	Severely dry
≤−2	Extremely dry

### Trend-free prewhitening

The purpose of trend-free prewhitening (TFPW) is to remove the influence of the autocorrelation of the data sequence on the results of the trend analysis^47^. The calculation steps are as follows. (a) The slope (*β*) of a trend in sample data is estimated using the approach proposed by Theil^48^ and Sen^49^. The original sample data *X*_*t*_ were unitized by dividing each value by the sample mean E (*X*_*t*_) prior to conducting the trend analysis. In this method, the mean of the new data *X*_*t*_ is equal to one, and the properties of the original sample data remain unchanged. If the slope is almost equal to zero, then it is not necessary to continue the trend analysis. However, if the slope differs from zero, then it is assumed to be linear, and the sample data are detrended as follows.

6 $${X}_{t}^{\text{'}}={X}_{t}-{T}_{t}={X}_{t}-\beta \cdot t$$

In addition, the slope *β* is calculated as follows.

7 $$\beta ={\rm{median}}\frac{{x}_{j}-{x}_{i}}{j-i}\,\forall \,j > i$$

b) The lag-1 serial correlation coefficient of sample data *x*_*i*_ (designated as *R*_*h*_) can be expressed as follows.

8 $${R}_{h}=\frac{\frac{1}{n}{\sum }_{i=1}^{n-1}({x}_{i}-\mu ({x}_{i}))\cdot ({x}_{i+1}-\mu ({x}_{i}))}{\frac{1}{n}{\sum }_{i=1}^{n}{({x}_{i}-\mu ({x}_{i}))}^{2}}$$

9 $$\mu ({x}_{i})=\frac{1}{n}\sum _{i=1}^{n}{x}_{i}$$

In the two-sided test, *R*_*h*_ was computed using the following equation at the 95% significance level^50^:

10 $$\frac{-1-1.96\sqrt{n-2}}{n-1} < {R}_{h}(95 \% ) < \frac{-1+1.96\sqrt{n-2}}{n-1}$$

where *n* is the sample size.

For the lag-1 serial correlation coefficient (*r*_1_) of the detrended series ${X}_{t}^{\text{'}}$, if *r*_1_ falls within the range calculated by Eq. (10), the sample data are considered to be serially independent. In this case, linear trend estimation, Spearman’s rho test and Mann-Kendall test methods are directly applied to ${X}_{t}^{\text{'}}$. Otherwise, the sample data ${Y}_{t}^{\text{'}}$ can be obtained by the following equation.

11 $${Y}_{t}^{\text{'}}={X}_{t}^{\text{'}}-{r}_{1}\cdot {X}_{t-1}^{\text{'}}$$

This prewhitening procedure after detrending the series is referred to as the trend-free prewhitening procedure. After applying the TFPW procedure, the residual series should be an independent series. c) The identified trend (T_t_) and the residual ${Y}_{t}^{\text{'}}$ are combined as follows.

12 $${Y}_{t}={Y}_{t}^{\text{'}}+{T}_{t}$$

The blended series (*Y*_*t*_) includes a trend and noise and is no longer influenced by the serial correlation. Then, the M-K test is applied to the blended series to assess the significance of the trend.

### Linear trend estimation

*x*_*i*_ denotes a climate variable with a sample size of *n*, and *t*_*i*_ denotes the time corresponding to *x*_*i*_. A linear regression equation can be established between *x*_*i*_ and *t*_*i*_ as follows.

13 $${x}_{i}=a+b{t}_{i},\,i=1,\,2,\,\ldots ,\,n$$

The above equation can be regarded as a special form of linear regression, where *a* is the regression constant and *b* is the regression coefficient. *a* and *b* can be estimated using the least squares method. The regression coefficient b indicates the trend of the climate variable. Thus, *b* > 0 indicates that the climate variable *x* increases with time, and *b* < 0 indicates the opposite trend. The value of *b* reflects the rate of increase or decrease, namely, the degree of the tendency. In general, *b* (*b* × 10) indicates the rate of the trend [units: mm/year or °C /year (mm/decade or °C/decade)], which can be used to quantitatively analyze the linear changes in climate elements.

### Spearman’s rho test

Spearman’s rho test is a non-parametric test commonly used to determine the trends of time series that are not normally distributed. This test can be expressed as follows:

14 $$D=1-\frac{[6{\sum }_{i=1}^{n}{(R({X}_{i})-i)}^{2}]}{n({n}^{2}-1)}$$

15 $${Z}_{D}=D\sqrt{\frac{n-2}{1-{D}^{2}}}$$

where *n* is the length of the time series; *R*(*X*_*i*_) is the rank of the time series at the observed value *X*_*i*_; and positive and negative values of *Z*_*D*_ indicate upward and downward trends, respectively. |*Z*_*D*_| > 2.08 rejects the hypothesis of no trend at the 5% significance level.

### Mann-Kendall trend test

In the Mann-Kendall (M–K) trend test^51, 52^, the null hypothesis *H*_0_ is that the time series data (*x*_1_, × _2_…, *x*_*n*_) comprise a sample of *n* independent and identically distributed random variables. The alternative hypothesis H_1_ is a bilateral test: for all *k*, *j* ≤ *n* and *k* ≠ *j* when *x*_*k*_ and *x*_*j*_ are differently distributed. The statistical variable *S* is calculated as follows:

16 $$S=\sum _{k=1}^{n-1}\sum _{j=k+1}^{n}Sgn({x}_{j}-{x}_{k})$$

where *Sgn*(*x*) is the sign function. The associated value is determined using the following equation.

17 $$Sgn({x}_{j}-{x}_{k})=\{\begin{array}{lll}+1 & {x}_{j}-{x}_{k} &  > 0\\ 0 & {x}_{j}-{x}_{k} & =0\\ -1 & {x}_{j}-{x}_{k} &  < 0\end{array}$$

*S* is normally distributed and has a mean of zero, and the variance is denoted as $Var=n(n-1)(2n+5)/18$. Whe*n n* > 10, the standard normal statistical variable *Z* can be calculated as follows.

18 $$Z=\{\begin{array}{cc}\frac{S-1}{\sqrt{Var(S)}} & S > 0\\ 0 & S=0\\ \frac{S+1}{\sqrt{Var(S)}} & S < 0\end{array}.$$

In the bilateral trend test, if *|Z| i*s ≥${Z}_{1-(\alpha /2)}$ at the given confidence level of *α*, the null hypothesis H_0_ is unacceptable, i.e., the time series exhibits a significant upward or downward trend at the confidence level of *α*. Specifically, if *Z* > 0, the series displays an upward or increasing trend, and if *Z* < 0, the series shows a downward or decreasing trend. If *|Z|* ≥  1.28, 1.64, or 2.32, the significant trend test is passed at 90%, 95%, or 99% confidence levels, respectively.

### Mann-Kendall abrupt change test

A rank series is constructed for time series *x* with a sample size of *n*.

19 $${s}_{k}=\sum _{i=1}^{k}{r}_{i}\,(k=2,3,\,\ldots ,\,n),$$

Notably, when *x*_*i*_ > *x*_*j*_, *r*_*i*_ = +1, and when *x*_*i*_ < *x*_*j*_, *r*_*i*_ = 0, where *j* = 1, 2, …, *i*.

The statistical variable is defined based on the assumption of an independent random time series:

20 $$U{F}_{K}=\frac{[{s}_{k}-\overline{{s}_{k}}]}{\sqrt{Var({s}_{k})}}\,(k=1,2,\ldots ,n)$$

where UF_1_ = 0, and $\overline{{s}_{k}}$ and Var(s_*k*_) are the mean and variance of the cumulative value *s*_*k*_, respectively. When ×_1_, ×_2_…, *x*_*n*_ are independent in the same continuous distribution, $\overline{{s}_{k}}$ and *Var*(*s*_*k*_) can be calculated as follows.

21 $$\{\begin{array}{c}\overline{{s}_{k}}=\frac{n(n+1)}{4}\\ Var({s}_{k})=\frac{n(n-1)(2n+5)}{72}\end{array}$$

UF_*i*_, a standard normal distribution, is a statistical series calculated based on the order of time series *x*. At a given significance level *α*, |UF_*i*_| > U_*α*_ indicates a significant trend in the series. The above calculation procedure is repeated in reverse order for time series x, and UB_*k*_ = −UF_*k*_ (k = *n*, *n*−1, …, 1), where UB_1_ = 0. Then, UF_*k*_ > 0 indicates an upward or increasing trend, UF_*k*_ < 0 indicates a downward or decreasing trend, and exceeding the critical line indicates a significant trend. If the two curves formed by UF_*k*_ and UB_*k*_ intersect and if the intersection appears between the boundary lines, the corresponding time of the intersection is the start time of an abrupt change.

### Rescaled range analysis

In this study, the rescaled range analysis (R/S) method was used to calculate the Hurst coefficient and predict and quantitatively describe the long-term correlation of the time series^53, 54^.

The time series U with length M is divided into [*M*/*N*] subsequences *u*_*i*_ (*i* = 2, 3…, [*M*/*N*]) with length N. The range of each subsequence can be calculated as follows:

22 $${R}_{u}=max{Z}_{u}-min{Z}_{u}$$

where *Z*_*u*_ is the sequential cumulative deviation in subsequence *u*_*i*_.

*R*_*u*_/*S*_*u*_ is the rescaled range of each subsequence, and *S*_*u*_ is the standard deviation of subsequence *u*_*i*_.

The logarithmic processing of the empirical Hurst formula (*R*_*N*_/*S*_*N*_ = *ωN* ^H^) can be expressed as follows:

23 $$lg({R}_{N}/{S}_{N})=HlgN+lg\omega $$

where *H* indicates the Hurst index and *ω* is a constant.

*H* has three value areas with different physical significance: (a) *H* ∈ (0, 0.5) indicates that the direction of the overall trend in the future is opposite that of the previous trend; (b) *H* ∈ (0.5, 1) indicates a time series with long-term positive autocorrelation, i.e., a change in the overall direction of the time series will reflect the previous trend; and (c) *H* = 0.5 indicates a completely uncorrelated series. For time series, the result of each time range is absolutely independent. Therefore, changes in time series are completely random.

## Electronic supplementary material

Dataset 1

Dataset 2

Dataset 3
